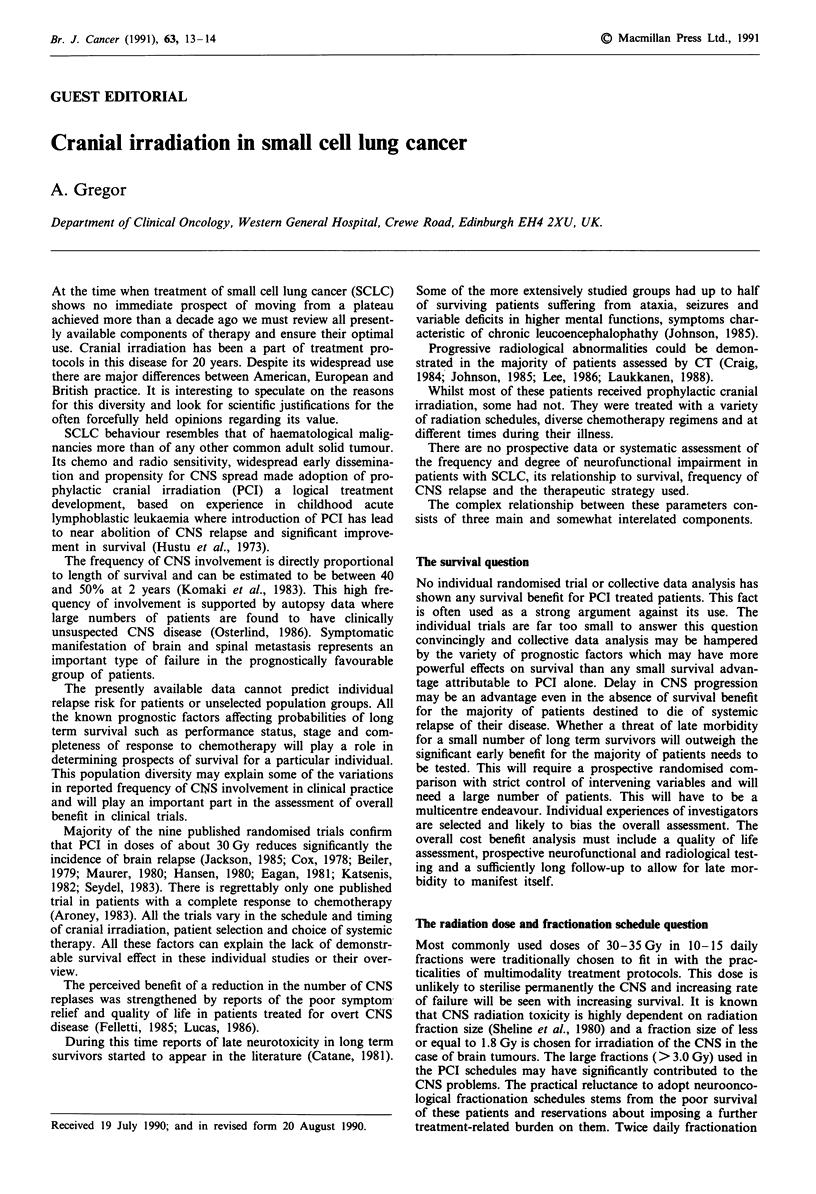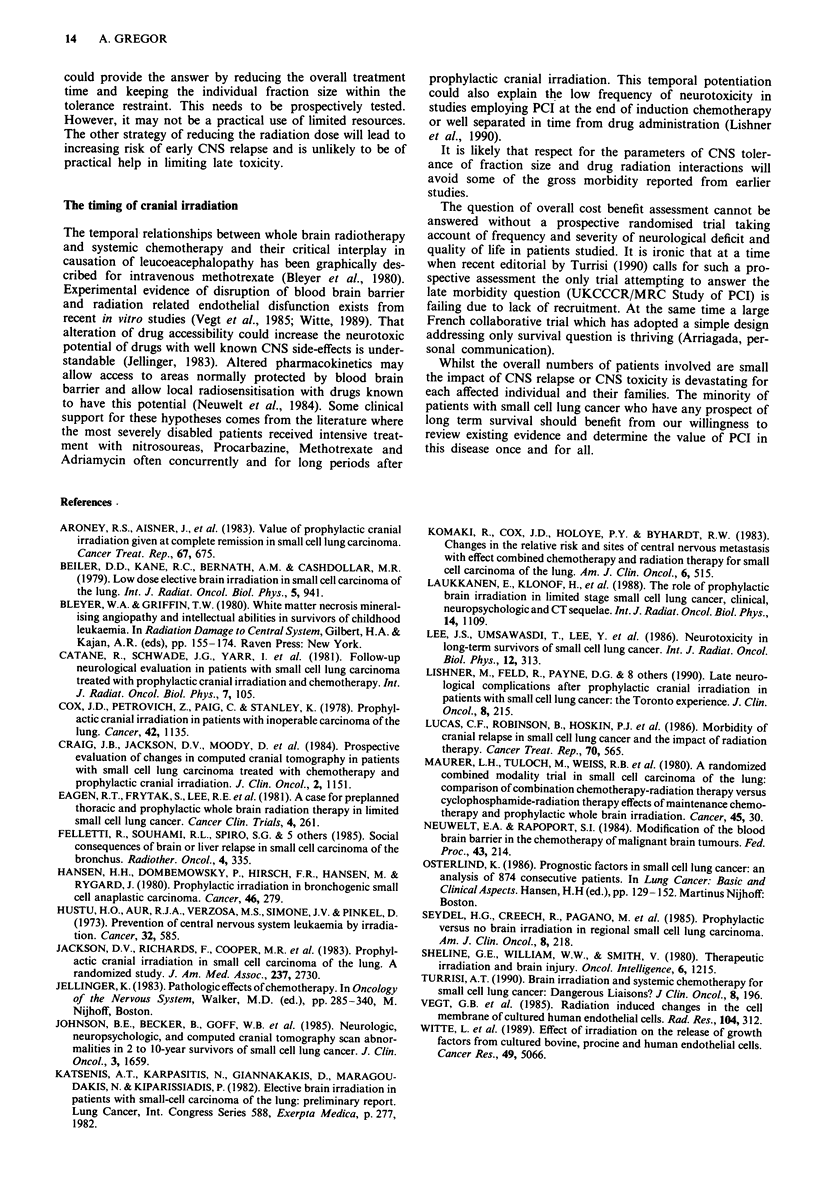# Cranial irradiation in small cell lung cancer.

**DOI:** 10.1038/bjc.1991.5

**Published:** 1991-01

**Authors:** A. Gregor


Br. J. Cancer (1991), 63, 13–14 © Macmillan Press Ltd., 1991

GUEST EDITORIAL

### Cranial irradiation in small cell lung cancer

A. Gregor

*Department of Clinical Oncology, Western General Hospital, Crewe Road, Edinburgh EH4 2XU, UK.*

At the time when treatment of small cell lung cancer (SCLC) shows no immediate prospect of moving from a plateau achieved more than a decade ago we must review all presently available components of therapy and ensure their optimal use. Cranial irradiation has been a part of treatment protocols in this disease for 20 years. Despite its widespread use there are major differences between American, European and British practice. It is interesting to speculate on the reasons for this diversity and look for scientific justifications for the often forcefully held opinions regarding its value.

SCLC behaviour resembles that of haematological malignancies more than of any other common adult solid tumour. Its chemo and radio sensitivity, widespread early dissemination and propensity for CNS spread made adoption of prophylactic cranial irradiation (PCI) a logical treatment development, based on experience in childhood acute lymphoblastic leukaemia where introduction of PCI has lead to near abolition of CNS relapse and significant improvement in survival (Hustu *et al.*, 1973).

The frequency of CNS involvement is directly proportional to length of survival and can be estimated to be between 40 and 50% at 2 years (Komaki *et al.*, 1983). This high frequency of involvement is supported by autopsy data where large numbers of patients are found to have clinically unsuspected CNS disease (Osterlind, 1986). Symptomatic manifestation of brain and spinal metastasis represents an important type of failure in the prognostically favourable group of patients.

The presently available data cannot predict individual relapse risk for patients or unselected population groups. All the known prognostic factors affecting probabilities of long term survival such as performance status, stage and completeness of response to chemotherapy will play a role in determining prospects of survival for a particular individual. This population diversity may explain some of the variations in reported frequency of CNS involvement in clinical practice and will play an important part in the assessment of overall benefit in clinical trials.

Majority of the nine published randomised trials confirm that PCI in doses of about 30 Gy reduces significantly the incidence of brain relapse (Jackson, 1985; Cox, 1978; Beiler, 1979; Maurer, 1980; Hansen, 1980; Eagan, 1981; Katsenis, 1982; Seydel, 1983). There is regrettably only one published trial in patients with a complete response to chemotherapy (Aroney, 1983). All the trials vary in the schedule and timing of cranial irradiation, patient selection and choice of systemic therapy. All these factors can explain the lack of demonstrable survival effect in these individual studies or their overview.

The perceived benefit of a reduction in the number of CNS replases was strengthened by reports of the poor symptom relief and quality of life in patients treated for overt CNS disease (Felletti, 1985; Lucas, 1986).

During this time reports of late neurotoxicity in long term survivors started to appear in the literature (Catane, 1981). Some of the more extensively studied groups had up to half of surviving patients suffering from ataxia, seizures and variable deficits in higher mental functions, symptoms characteristic of chronic leucoencephalophathy (Johnson, 1985).

Progressive radiological abnormalities could be demonstrated in the majority of patients assessed by CT (Craig, 1984; Johnson, 1985; Lee, 1986; Laukkanen, 1988).

Whilst most of these patients received prophylactic cranial irradiation, some had not. They were treated with a variety of radiation schedules, diverse chemotherapy regimens and at different times during their illness.

There are no prospective data or systematic assessment of the frequency and degree of neurofunctional impairment in patients with SCLC, its relationship to survival, frequency of CNS relapse and the therapeutic strategy used.

The complex relationship between these parameters consists of three main and somewhat interelated components.

### The survival question

No individual randomised trial or collective data analysis has shown any survival benefit for PCI treated patients. This fact is often used as a strong argument against its use. The individual trials are far too small to answer this question convincingly and collective data analysis may be hampered by the variety of prognostic factors which may have more powerful effects on survival than any small survival advantage attributable to PCI alone. Delay in CNS progression may be an advantage even in the absence of survival benefit for the majority of patients destined to die of systemic relapse of their disease. Whether a threat of late morbidity for a small number of long term survivors will outweigh the significant early benefit for the majority of patients needs to be tested. This will require a prospective randomised comparison with strict control of intervening variables and will need a large number of patients. This will have to be a multicentre endeavour. Individual experiences of investigators are selected and likely to bias the overall assessment. The overall cost benefit analysis must include a quality of life assessment, prospective neurofunctional and radiological testing and a sufficiently long follow-up to allow for late morbidity to manifest itself.

### The radiation dose and fractionation schedule question

Most commonly used doses of 30–35 Gy in 10–15 daily fractions were traditionally chosen to fit in with the practicalities of multimodality treatment protocols. This dose is unlikely to sterilise permanently the CNS and increasing rate of failure will be seen with increasing survival. It is known that CNS radiation toxicity is highly dependent on radiation fraction size (Sheline *et al.*, 1980) and a fraction size of less or equal to 1.8 Gy is chosen for irradiation of the CNS in the case of brain tumours. The large fractions (> 3.0 Gy) used in the PCI schedules may have significantly contributed to the CNS problems. The practical reluctance to adopt neurooncological fractionation schedules stems from the poor survival of these patients and reservations about imposing a further treatment-related burden on them. Twice daily fractionation

Received 19 July 1990; and in revised form 20 August 1990.

could provide the answer by reducing the overall treatment time and keeping the individual fraction size within the tolerance restraint. This needs to be prospectively tested. However, it may not be a practical use of limited resources. The other strategy of reducing the radiation dose will lead to increasing risk of early CNS relapse and is unlikely to be of practical help in limiting late toxicity.

### The timing of cranial irradiation

The temporal relationships between whole brain radiotherapy and systemic chemotherapy and their critical interplay in causation of leucoeacephalopathy has been graphically described for intravenous methotrexate (Bleyer *et al.*, 1980). Experimental evidence of disruption of blood brain barrier and radiation related endothelial disfunction exists from recent *in vitro* studies (Vegt *et al.*, 1985; Witte, 1989). That alteration of drug accessibility could increase the neurotoxic potential of drugs with well known CNS side-effects is understandable (Jellinger, 1983). Altered pharmacokinetics may allow access to areas normally protected by blood brain barrier and allow local radiosensitisation with drugs known to have this potential (Neuwelt *et al.*, 1984). Some clinical support for these hypotheses comes from the literature where the most severely disabled patients received intensive treatment with nitrosoureas, Procarbazine, Methotrexate and Adriamycin often concurrently and for long periods after prophylactic cranial irradiation. This temporal potentiation could also explain the low frequency of neurotoxicity in studies employing PCI at the end of induction chemotherapy or well separated in time from drug administration (Lishner *et al.*, 1990).

It is likely that respect for the parameters of CNS tolerance of fraction size and drug radiation interactions will avoid some of the gross morbidity reported from earlier studies.

The question of overall cost benefit assessment cannot be answered without a prospective randomised trial taking account of frequency and severity of neurological deficit and quality of life in patients studied. It is ironic that at a time when recent editorial by Turrisi (1990) calls for such a prospective assessment the only trial attempting to answer the late morbidity question (UKCCCR/MRC Study of PCI) is failing due to lack of recruitment. At the same time a large French collaborative trial which has adopted a simple design addressing only survival question is thriving (Arriagada, personal communication).

Whilst the overall numbers of patients involved are small the impact of CNS relapse or CNS toxicity is devastating for each affected individual and their families. The minority of patients with small cell lung cancer who have any prospect of long term survival should benefit from our willingness to review existing evidence and determine the value of PCI in this disease once and for all.

### References

ARONEY, R.S., AISNER, J., *et al.* (1983). Value of prophylactic cranial irradiation given at complete remission in small cell lung carcinoma. *Cancer Treat. Rep.*, **67**, 675.

BEILER, D.D., KANE, R.C., BERNATH, A.M. & CASHDOLLAR, M.R. (1979). Low dose elective brain irradiation in small cell carcinoma of the lung. *Int. J. Radiat. Oncol. Biol. Phys.*, **5**, 941.

BLEYER, W.A. & GRIFFIN, T.W. (1980). White matter necrosis mineralising angiopathy and intellectual abilities in survivors of childhood leukaemia. In *Radiation Damage to Central System*, Gilbert, H.A. & Kajan, A.R. (eds), pp. 155–174. Raven Press: New York.

CATANE, R., SCHWADE, J.G., YARR, I. *et al.* (1981). Follow-up neurological evaluation in patients with small cell lung carcinoma treated with prophylactic cranial irradiation and chemotherapy. *Int. J. Radiat. Oncol. Biol. Phys.*, **7**, 105.

COX, J.D., PETROVICH, Z., PAIG, C. & STANLEY, K. (1978). Prophylactic cranial irradiation in patients with inoperable carcinoma of the lung. *Cancer*, **42**, 1135.

CRAIG, J.B., JACKSON, D.V., MOODY, D. *et al.* (1984). Prospective evaluation of changes in computed cranial tomography in patients with small cell lung carcinoma treated with chemotherapy and prophylactic cranial irradiation. *J. Clin. Oncol.*, **2**, 1151.

EAGEN, R.T., FRYTAK, S., LEE, R.E. *et al.* (1981). A case for preplanned thoracic and prophylactic whole brain radiation therapy in limited small cell lung cancer. *Cancer Clin. Trials*, **4**, 261.

FELLETTI, R., SOUHAMI, R.L., SPIRO, S.G. & 5 others (1985). Social consequences of brain or liver relapse in small cell carcinoma of the bronchus. *Radiother. Oncol.*, **4**, 335.

HANSEN, H.H., DOMBEMOWSKY, P., HIRSCH, F.R., HANSEN, M. & RYGARD, J. (1980). Prophylactic irradiation in bronchogenic small cell anaplastic carcinoma. *Cancer*, **46**, 279.

HUSTU, H.O., AUR, R.J.A., VERZOSA, M.S., SIMONE, J.V. & PINKEL, D. (1973). Prevention of central nervous system leukaemia by irradiation. *Cancer*, **32**, 585.

JACKSON, D.V., RICHARDS, F., COOPER, M.R. *et al.* (1983). Prophylactic cranial irradiation in small cell carcinoma of the lung. A randomized study. *J. Am. Med. Assoc.*, **237**, 2730.

JELLINGER, K. (1983). Pathologic effects of chemotherapy. In *Oncology of the Nervous System*, Walker, M.D. (ed.), pp. 285–340, M. Nijhoff, Boston.

JOHNSON, B.E., BECKER, B., GOFF, W.B. *et al.* (1985). Neurologic, neuropsychologic, and computed cranial tomography scan abnormalities in 2 to 10-year survivors of small cell lung cancer. *J. Clin. Oncol.*, **3**, 1659.

KATSENIS, A.T., KARPASITIS, N., GIANNAKAKIS, D., MARAGOUDAKIS, N. & KIPARISSIADIS, P. (1982). Elective brain irradiation in patients with small-cell carcinoma of the lung: preliminary report. Lung Cancer, Int. Congress Series 588, *Exerpta Medica*, p. 277, 1982.

KOMAKI, R., COX, J.D., HOLOYE, P.Y. & BYHARDT, R.W. (1983). Changes in the relative risk and sites of central nervous metastasis with effect combined chemotherapy and radiation therapy for small cell carcinoma of the lung. *Am. J. Clin. Oncol.*, **6**, 515.

LAUKKANEN, E., KLONOF, H., *et al.* (1988). The role of prophylactic brain irradiation in limited stage small cell lung cancer, clinical, neuropsychologic and CT sequelae. *Int. J. Radiat. Oncol. Biol. Phys.*, **14**, 1109.

LEE, J.S., UMSAWASDI, T., LEE, Y. *et al.* (1986). Neurotoxicity in long-term survivors of small cell lung cancer. *Int. J. Radiat. Oncol. Biol. Phys.*, **12**, 313.

LISHNER, M., FELD, R., PAYNE, D.G. & 8 others (1990). Late neurological complications after prophylactic cranial irradiation in patients with small cell lung cancer: the Toronto experience. *J. Clin. Oncol.*, **8**, 215.

LUCAS, C.F., ROBINSON, B., HOSKIN, P.J. *et al.* (1986). Morbidity of cranial relapse in small cell lung cancer and the impact of radiation therapy. *Cancer Treat. Rep.*, **70**, 565.

MAURER, L.H., TULOCH, M., WEISS, R.B. *et al.* (1980). A randomized combined modality trial in small cell carcinoma of the lung: comparison of combination chemotherapy-radiation therapy versus cyclophosphamide-radiation therapy effects of maintenance chemotherapy and prophylactic whole brain irradiation. *Cancer*, **45**, 30.

NEUWELT, E.A. & RAPOPORT, S.I. (1984). Modification of the blood brain barrier in the chemotherapy of malignant brain tumours. *Fed. Proc.*, **43**, 214.

OSTERLIND, K. (1986). Prognostic factors in small cell lung cancer: an analysis of 874 consecutive patients. In *Lung Cancer: Basic and Clinical Aspects*. Hansen, H.H (ed.), pp. 129–152. Martinus Nijhoff: Boston.

SEYDEL, H.G., CREECH, R., PAGANO, M. *et al.* (1985). Prophylactic versus no brain irradiation in regional small cell lung carcinoma. *Am. J. Clin. Oncol.*, **8**, 218.

SHELINE, G.E., WILLIAM, W.W., & SMITH, V. (1980). Therapeutic irradiation and brain injury. *Oncol. Intelligence*, **6**, 1215.

TURRISI, A.T. (1990). Brain irradiation and systemic chemotherapy for small cell lung cancer: Dangerous Liaisons? *J Clin. Oncol.*, **8**, 196.

VEGT, G.B. *et al.* (1985). Radiation induced changes in the cell membrane of cultured human endothelial cells. *Rad. Res.*, **104**, 312.

WITTE, L. *et al.* (1989). Effect of irradiation on the release of growth factors from cultured bovine, procine and human endothelial cells. *Cancer Res.*, **49**, 5066.